# Propofol Upregulates MicroRNA-30b to Inhibit Excessive Autophagy and Apoptosis and Attenuates Ischemia/Reperfusion Injury In Vitro and in Patients

**DOI:** 10.1155/2022/2109891

**Published:** 2022-03-30

**Authors:** Zhiqi Lu, Jiaojiao Shen, Xubin Chen, Zhihua Ruan, Weihua Cai, Shuyun Cai, Minjun Li, Yihui Yang, Jian Mo, Guixi Mo, Yan Lu, Jing Tang, Liangqing Zhang

**Affiliations:** ^1^Department of Anesthesiology, Affiliated Hospital of Guangdong Medical University, Zhanjiang, Guangdong, China 524001; ^2^Department of Anesthesiology, Hainan General Hospital, Hainan Affiliated Hospital of Hainan Medical University, Haikou, Hainan, China 570311; ^3^Department of Oral and Maxillofacial Surgery, Hainan General Hospital, Hainan Affiliated Hospital of Hainan Medical University, Haikou, Hainan, China 570311

## Abstract

Evidence reveals that propofol protects cells via suppressing excessive autophagy induced by hypoxia/reoxygenation (H/R). Previously, we found in a genome-wide microRNA profile analysis that several autophagy-related microRNAs were significantly altered during the process of H/R in the presence or absence of propofol posthypoxia treatment (P-PostH), but how these microRNAs work in P-PostH is still largely unknown. Here, we found that one of these microRNAs, microRNA-30b (miR-30b), in human umbilical vein endothelial cells (HUVECs) was downregulated by H/R treatment but significantly upregulated by 100 M propofol after H/R treatment. miR-30b showed similar changes in open heart surgery patients. By dual-luciferase assay, we found that Beclin-1 is the direct target of miR-30b. This conclusion was also supported by knockdown or overexpression of miR-30b. Further studies showed that miR-30b inhibited H/R-induced autophagy activation. Overexpression or knockdown of miR-30b regulated autophagy-related protein gene expression in vitro. To clarify the specific role of propofol in the inhibition of autophagy and distinguish the induction of autophagy from the damage of autophagy flux, we used bafilomycin A1. LC3-II levels were decreased in the group treated with propofol combined with bafilomycin A1 compared with the group treated with bafilomycin A1 alone after hypoxia and reoxygenation. Moreover, HUVECs transfected with Ad-mCherry-GFP-LC3b confirmed the inhibitory effect of miR-30b on autophagy flux. Finally, we found that miR-30b is able to increase the cellular viability under the H/R condition, partially mimicking the protective effect of propofol which suppressed autophagy via enhancing miR-30b and targeting Beclin-1. Therefore, we concluded that propofol upregulates miR-30b to repress excessive autophagy via targeting Beclin-1 under H/R condition. Thus, our results revealed a novel mechanism of the protective role of propofol during anesthesia. *Clinical Trial Registration Number*. This trial is registered with ChiCTR-IPR-14005470. The name of the trial register: Propofol Upregulates MicroRNA-30b to Repress Beclin-1 and Inhibits Excessive Autophagy and Apoptosis.

## 1. Introduction

Ischemia reperfusion (I/R) leads to a significant inflammatory response which in turn may cause widespread cellular injury and microvascular dysfunction [1–3]. The mechanisms that contribute to the injuries include the increases in the activation of multiple genes involved in apoptosis pathway, Ca^2+^ concentration, and induction of reactive oxygen species (ROS) sources during reperfusion. All these accumulatively lead to the overactivation of autophagy and cell apoptosis [2–7].

Some studies showed that sevoflurane preconditioning has a protective effect on cardiac ischemic tissues [8, 9]. Chappell et al. found that the effect of cardiac sevoflurane preconditioning has been related to the endothelial protection and to the beneficial effects on inflammatory response [10–12]. In addition, as an intravenous anesthetic with rapid action, short half-life and high recovery quality, propofol is widely used in clinical anesthesia [13–15]. The chemical structure of propofol is similar to that of *α*-tocopherol (vitamin E), and it has antioxidant properties. Accumulating evidence supported that the anesthetic agents propofol, when used as a protective strategy, can reduce the extent of cardio, lung, or brain injuries [4, 5, 16–18]. It has been shown to protect neuronal cells from hypoxia-reoxygenation (H/R) injury, possibly via an antioxidant action under hypoxic conditions [19, 20]. Some reports indicated that propofol may inhibit I/R-activated autophagic cell death through affecting the expressions of autophagy-related genes to prevent ischemia or hypoxia reoxygenation injury [7, 16, 21–24], but the mechanism by which propofol counteracts the cellular and tissue damages remains largely unknown.

Autophagy is the process of lysosomal degradation of damaged proteins and organelles [25]. Studies have shown that the beclin-1 protein encoded by the Beclin1 gene is a major regulator of autophagy in mammalian cells [26]. Beclin-1 is involved in the early stage of autophagy as part of a lipid kinase complex that stimulates the formation of the isolation membrane, a double-membrane structure that engulfs cytoplasmic material to form the autophagosomes [27]. Beclin-1 plays an important role in the development, tumorigenesis, and neurodegeneration [28]. Recent literature suggested that environmental factors such as nutritional deficiency and virus infection can stimulate Beclin-1 expression, but Beclin-1 expression induces autophagy and causes cell death [29, 30]. At the same time, under certain conditions, excessive and long-term autophagy may inhibit cell proliferation and even accelerate the death of cardiomyocytes [31–33]. These findings suggest that cardiac ischemia-reperfusion may cause autophagy apoptosis and cell damage.

MicroRNAs (miRNAs) are small noncoding RNA gene products about 22 nucleotide long that negatively regulate genes in a cell via translation inhibition of their target mRNAs [34]. The expression of microRNA is closely related to metabolic or physiological processes as well as many diseases [35–39]. In our previous study, we found that propofol reduces H/R-induced autophagic cell death through differentially regulating a group of miRNAs, six of which may target autophagy genes to repress overactivation of autophagy and therefore maintain the cell viability under H/R condition. The levels of hsa-miR-30b, hsa-miR-20b, hsa-miR-196a, and hsa-miR-374b were upregulated in the propofol-treated group with the HR only group, while hsa-let-7e and hsa-miR-15b showed an opposite expression pattern [7]. Beclin-1 and ATG5 were also shown to be the target genes of hsa-miR-30b [40], which was confirmed by our study, but the detail mechanism remains unknown.

We set up a hypothesis that propofol may change the expression of microRNAs to regulate autophagy-related protein and then inhibit autophagic cell death under hypoxia-reoxygenation conditions by sevoflurane preconditioning plus propofol postconditioning model.

## 2. Materials and Methods

The study was approved by the Hospital Ethical Committee. All clinical trials involving assignment of patients to treatment groups were registered prior to patient enrollment. The registry is Chinese Clinical Trial Registry; Clinical Trial Registration Number: ChiCTR-IPR-14005470; principal investigator's name: Liangqing Zhang, and the name of the trial register: Propofol Upregulates MicroRNA-30b to Repress Beclin-1 and Inhibits Excessive Autophagy and Apoptosis.

### 2.1. Cell Culture and Different Concentrations of Propofol Posthypoxia Treatment H/R Model In Vitro

The human umbilical vein endothelial cells (HUVECs) were purchased from Shanghai Cell Bank and cultured at 37°C in a 95% O_2_ and 5% CO_2_ humidified atmosphere in Dulbecco's modified Eagle's medium (DMEM) supplemented with 10% fetal bovine serum, 100 *μ*g/mL streptomycin, and 100 IU/mL penicillin (GIBCO Laboratories, Grand Island, New York, USA). HUVECs were treated with different concentrations of propofol (0–150 *μ*mol/L) in reoxygenation episode for 4 hours after 12 hours hypoxia. Or after 12 hours hypoxia, HUVECs were treated with the autophagy inhibitor 3-methyladenine (10 mmol/L) or bafilomycin A1 (Baf; 100 nmol/L) for 30 min or the pan caspase inhibitor Z-VAD-FMK (20 *μ*mol/L) for 1 h.

### 2.2. MicroRNA Transfection and H/R Model In Vitro

The HUVECs were seeded on 6-well plates in a DMEM (Dulbecco's modified Eagle medium, GIBCO), L-glutamine, and sodium pyruvate medium until 70% of confluence prior to transfection. The cells are transfected with 50 nM miR-30b mimic, 50 nM miR-30b negative control (NC), 100 nM miR-30b inhibitor, and 100 nM miR-30b inhibitor negative control (NC inhibitor) by a transfection kit (Guangzhou RiboBio Co., Ltd.). 24 hours after transfection, the cells were treated by 12 hours hypoxia in glucose and serum-free DMEM and then 4 hours reoxygenation in high glucose and 10% serum DMEM. At the same time, propofol posthypoxia treatment groups administrated 100 *μ*mol/L propofol on reoxygenation. The selection of 100 *μ*mol/L propofol was based on our preliminary study and previous study [7] which showed that posthypoxic cell viability was the highest concomitant with most apparent antiautophagy effects when propofol was applied at 100 *μ*mol/L than at any other concentrations ranging from 25 to 150 *μ*mol/L.

### 2.3. Human Study

The study has been approved by the Hospital Ethical Committee. It was carried out in the Affiliated Hospital of Guangdong Medical College (Zhanjiang, China). All procedures performed in studies involving human participants were in accordance with the ethical standards of the Affiliated Hospital of Guangdong Medical College and with the 1964 Helsinki declaration. The participating patients enrolled were well informed, and consent documents were signed. Forty patients, with ASA physical status III scheduled for mitral valve replacement surgery, were randomly allocated to the control group and propofol group (*n* = 20 per group). Eligible subjects were 20 to 65 years old. Myocardial relevant enzyme did not increase 24 hours before the operation. Ejection fraction should exceed 0.4. Standard monitoring was established including five-lead electrocardiography, invasive arterial pressure (radial artery cannulation), heart rate, central venous pressure (CVP), nasopharyngeal temperature, peripheral oxygen saturation by pulse oximetry, capnography, and bispectral index by electrodes monitor (BIS, Aspect Medical Systems). All the patients were premedicated with 0.1 mg/kg morphine and 0.3 mg scopolamine administered intramuscularly 1 h before anesthesia, which was induced with intravenous midazolam (1-2 mg), etomidate (0.3 mg/kg), sufentanil (2 *μ*g/kg), and pipecuronium (0.08 mg/kg). In the control group, anesthesia was maintained with 0.5-2% sevoflurane before and after cardiopulmonary bypass (CPB) and supplemental sufentanil and pipecuronium to adjust the BIS between 40 and 60. Midazolam was dosed continuously at 0.1 mg/kg/h during and after CPB. Patients in the propofol group were then administrated 4-6 mg/kg/h propofol during and after CPB, and sevoflurane was used at other times beyond CPB period in the same way as in the control group. The right auricle of the heart tissue (0.5 cm in diameter per sample) was collected at three time points in each patient, respectively (T_1_: the superior vena cave intubation when the heart was in normal situation; T_2_: 15 minutes after cardiac arrest when the heart was during ischemia period; and T_3_: 10 minutes after heart restated beating when the heart was in the reperfusion period). Western blot was used to assess the level of Beclin-1 between the control group and propofol group, and then, qRT-PCR was performed to confirm the expression of miR-30b. Besides, 5 ml blood sample was, respectively, collected from the median cubital vein before anesthesia induction (T0), 15 minutes before cardiopulmonary bypass (T1), 15 minutes after cardiac arrest (T2), 10 minutes and 1 hour and after the heart restarted beating (T3 and T4), and 24 h after operation (T5). The blood was centrifuged at 3000 g/min for 15 min at 4°C, and the supernate was collected to assess the changes of perioperative myocardial enzyme (including TNT-HS and CK-MB). If necessary, inotropes (dobutamine, dopamine, epinephrine, nitroglycerin, or in combination) were used. The indication for inotrope administration was a mean radial arterial blood pressure less than 60 mmHg as reported [41].

### 2.4. Western Blotting Analysis

Cell lysates were dissolved in RIPA buffer (50 mM Tris HCl, pH 8, 150 mM NaCl, 1% Nonidet P-40, 0.1% SDS, and 1% Triton X-100 plus proteinase inhibitors; Sigma). Protein concentration was determined by Bradford assay, and the samples containing 30 *μ*g were separated by 8-15% SDS-PAGE and blotted onto a polyvinylidene difluoride microporous membrane (Millipore, Billerica, Mass., USA). 5% nonfat milk in 20 mM Tris HCl, 150 mM NaCl, and 0.05% Tween 20 was used to block the membrane for 2 hours at room temperature. Membranes were incubated at 4°C overnight with a 1 : 1,000 dilution of primary antibody and then washed and revealed using secondary antibodies with horseradish peroxidase conjugate (1 : 50000, 2 h; Earth), exposed to enhanced chemiluminescence reagents. Densitometric analysis was performed to quantify the signal intensity. The primary antibody were anti-Beclin-1 (1 : 1000; Cell Signaling Technology), anti-p62 (1 : 1000; MBL), Bax (1 : 1000; Proteintech), Bcl-2 (1 : 1000; Proteintech), GAPDH (1 : 2000; Santa Cruz, sc-25778), anti-beta-actin antibody (Santa Cruz, sc-47778), anti-LC3B polyclonal antibody (Novous, NB100-2220), and anti-LC3 polyclonal antibody (MBL, PM152-3).

### 2.5. qRT-PCR Gene Expression Analysis

The expressions of miR-30b were determined using real-time reverse transcriptase polymerase chain reaction (qRT-PCR) method. Total RNA was extracted from the cells and right auricle heart tissue in each group and reverse-transcribed with the cDNA Synthesis Kit RevertAid (Thermo, USA). Total RNA was purified by means of TRIzol reagent (TAKARA, 9109). The qRT-PCR was run using SYBR Green reagents (Tiangen, China). The primer sequences used are as follows: for has-miR-30b, 5′-CGCTGTAAACATCCTACACTCA-3′ (forward) and 5′-GCAGGGTCCGAGGTATTC-3′ (reverse) and for U6, 5′-CTCGCTTCGGCAGCACATATACT-3′ (forward) and 5′-CGAATTTGCGTGTCATCCTTGCG-3′ (reverse). The relative mRNA quantity was determined by 2-△△ct method. Real-time PCR was using a LightCycler^⑧^480 sequence detector system (Roche Applied Sciences).

### 2.6. Luciferase Activity Assay

Luciferase reporter plasmids were reconstructed by inserting target fragments into pmirGLO Dual-Luciferase miRNA Target Expression Vectors (dual-luciferase: firefly luciferase and renilla luciferase). The HEK293T cells were cultured onto 96-well plates at 10000 cells per well overnight in DMEM-H medium (Gibco, 12800017) with 10% fetal bovine serum and then cotransfected with plasmids of dual-luciferase reporters and miRNA mimics or NC, detecting dual-luciferase activity by using the Dual-Glo Luciferase Assay System Kit (Promega, E2920) according to the instructions. Firefly luciferase activity was normalized to renilla luciferase activity.

### 2.7. Cell Viability Assay

CCK-8 Kit (Dojindo Co., Japan) was used to evaluate cell viability. The cells were plated at 1∗10^3^ − 1∗10^5^ cells per well in 96-well plates. The cells in the miR-30b group, microRNA-30b negative control (NC) group, miR-30b inhibitor group, and micR-30b inhibitor negative control (NC inhibitor) group were transfected with relevant microRNAs, respectively, and then subjected to H/R and 100 *μ*mol/L propofol posthypoxia treatment. The cells in the 6 wells were repeated to the same measure every groups. Finally, the cells were added to the medium containing 10% CCK-8, reacted at 37°C for 2 hours, and was read at 450 nm by a multimode microplate reader (BioTek, VT, USA). The cellular viability (%) was calculated using the formula ∗100% where As indicates the absorbance of the well containing supernatant from exposure or sham-exposure dishes; Ac indicates the absorbance of the well containing supernatant from the normal control; and Ab indicates the absorbance of the well containing culture medium with 10% CCK-8 solution.

### 2.8. RNA Transfection

Transfection of RNA was performed using Lipofectamine RNAi MAX Reagent according to the manufacturer's instructions. And RNA is as follows: hsa-miR-30b mimics (5′-UGUAAACAUCCUACACUCAGCU-3′; 5′-CUGAGUGUAGGAUGUUUACAUU-3′), negative control (NC) (5′-UUCUCCGAACGUGUCACGUTT-3′; 5′-ACGUGACACGUUCGGAGAATT-3′), hsa-miR-30b inhibitor (5′-AGCUGAGUGUAGGAUGUUUACA-3′), and miRNA inhibitor N.C. (NC inhibitor) (5′-CAGUACUUUUGUGUAGUACAA-3′) (GenePharma shanghai, China). The cells were transfected 24 hours before the next experiment.

### 2.9. Adenovirus Transfection (Ad-mCherry-GFP-LC3B)

HUVECs were cultured to 60% confluence and transfected with Ad-mCherry-GFP-LC3B (Beyotime Biotechnology) for 24 h. And then, HUVECs were fixed in 4% paraformaldehyde, subjected to 4′,6-diamidino-2-phenylindole (DAPI) staining to detect the nucleus. After treatment, the mCherry-GFP-LC3B fusion protein was visualized with the fluorescence microscope (Olympus FV1000, Tokyo, Japan). Yellow (merge of GFP signal and RFP signal) puncta represented early autophagosomes, while red (RFP signal alone) puncta indicated late autolysosomes. Autophagic flux was analyzed by the color change of GFP/RFP.

### 2.10. Statistical Analysis

Statistical analysis was performed with SPSS software, version 17.0 (SPSS, Inc., Chicago, IL, USA). All experiments were then done independently at least three times. Data are presented as means ± SD. Between-group variance in vitro was analyzed by a one-way ANOVA. In addition, we analyzed the variance between-group and within each group in vivo using ANOVA for repeated measurements. A *p* value less than 0.05 was considered statistically significantly different.

## 3. Results

**Figure 1:**
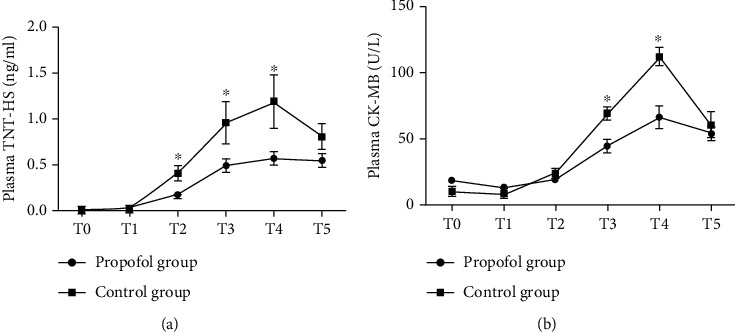
The change of myocardial enzymes during perioperative period. (a) The change of plasma TNT-HS between two groups in the perioperative period. (b) The change of plasma CK-MB between two groups in the perioperative period. Mean ± SD of 3 independent trials. ^∗^*p* < 0.05, vs. the control group. Abbreviations: T_0_: before anesthesia induction; T_1_: 15 min before cardiopulmonary bypass; T_2_: 15 min after cardiac arrest; T_3_: 10 minutes after heart restated beating; T_4_: 1 h after heart restated beating; T_5_: 24 h after operation; TNT-HS: sensitive troponin T; CK-MB: creatine kinase isoenzyme muscle/brain.

There were no significant differences in the patients' baseline characteristics and perioperative adverse reactions between two groups (*p* > 0.05) (Tables 1 and 2).  Table 3 shows the specific clinical data of patients during perioperative period. It can be seen that the heart rate and mean cardiac arterial pressure of patients at T_3_ and T_4_ is significantly different. We then measured the level of changes in markers of myocardial injury at each stage and found significant differences between T_3_ and T_4_ (Figures 1(a) and 1(b)). As shown in Table 2, perioperative adverse reactions were observed and recorded in detail, such as bradycardia, respiratory depression, and hypotension. Heart rate (HR) <50 beats/min was defined as bradycardia; mean arterial pressure (MAP) <60 mmHg was defined as hypotension. Perioperative hypotension and bradycardia were treated with epinephrine and atropine, respectively. Three patients in the control group and one in the propofol group experienced perioperative bradycardia and were treated with atropine (Table 2). If adverse reactions occur in the process of operation, record the time of occurrence at any time, and make relevant treatment according to its severity; if affecting the experimenter, the experimental group will be excluded.

### 3.1. Propofol Inhibits Autophagy in H/R (I/R) Models In Vitro or In Vivo

Perioperative clinical data showed that sensitive troponin T (TNT-HS) at T_2_, T_3_, and T_4_ and creatine kinase isoenzyme muscle/brain (CK-MB) at T_3_ and T_4_ in the propofol group were lower than the control group (*p* < 0.05), indicating that the release level of myocardial injury markers in the propofol group was lower than that in the control group (Figures 1(a) and 1(b)). Some reports indicated that propofol may inhibit I/R-activated autophagic cell death through affecting the expressions of autophagy-related genes to prevent ischemia or hypoxia reoxygenation injury [7, 16, 21–24], but the mechanism by which propofol counteracts the cellular and tissue damages remains largely unknown.

**Figure 2:**
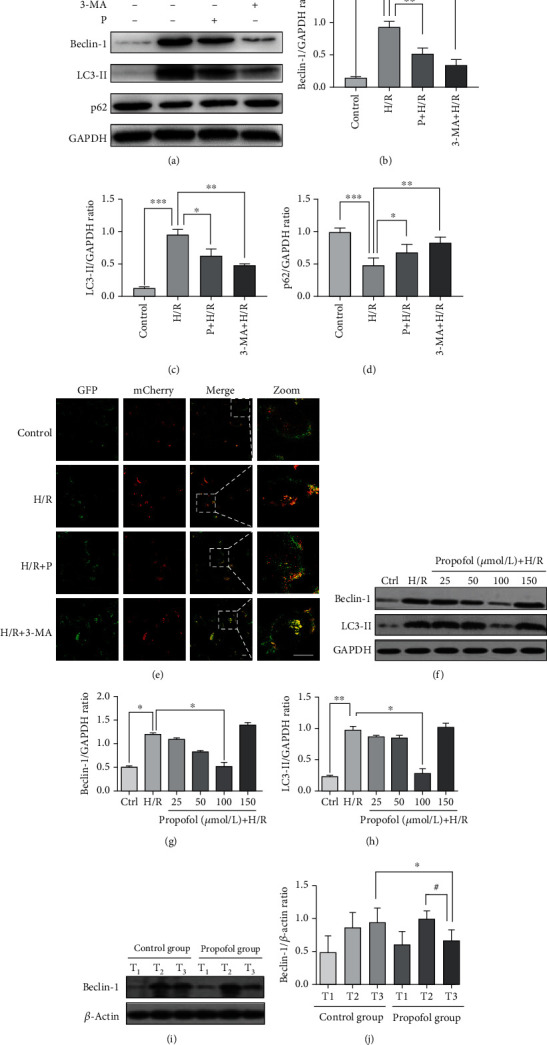
Propofol inhibits autophagy in H/R (I/R) models in vitro or in vivo. (a–d) The PI3K inhibitor 3-methyladenine (10 mM) was used to ascertain the activation of autophagy. ^∗^*p* < 0.05, vs. the H/R group. ^∗∗^*p* < 0.01, vs. the H/R group. ^∗∗∗^*p* < 0.001, vs. the H/R group. (e) HUVECs were transduced with Ad-mRFP-GFP-LC3B and then treated with 100 *μ*mol/L propofol or 10 mmol/L 3-MA after H/R. Representative images of fluorescent LC3 puncta. Yellow puncta: red puncta overlaid with green puncta and red puncta: autolysosomes. Scale bar = 20 *μ*m. (f–h) The cells were postconditioned at the very onset of reoxygenation with increasing concentrations of propofol (0–150 *μ*mol/L) for 4 h after 12 h of hypoxia. Expression of autophagy-related proteins in control group, H/R injury group, and propofol posthypoxia treatment groups. ^∗^*p* < 0.05, vs. the H/R group. ^∗∗^*p* < 0.01, vs. the H/R group. (i–j) The level of autophagy-related proteins Beclin-1 was examined in the control group (patients treated with midazolam and sevoflurane during reperfusion) and propofol group (patients treated with propofol and sevoflurane during reperfusion). ^∗^*p* < 0.05, T_3_ in the propofol group vs. T_3_ in the control group. ^#^*p* < 0.05, T_3_ in the propofol group vs. T_2_ in the propofol group. Mean ± SD of 3 independent trials. Abbreviations: H/R: hypoxia/re-oxygenation; P: propofol; 3-MA: 3-methyladenine; Ctrl or C: the control group; T_1_: 15 min before cardiopulmonary bypass; T_2_: 15 min after cardiac arrest; T_3_: 10 minutes after heart restated beating.

We first verified that H/R treatment induced autophagy in HUVECs at the cellular level. The levels of autophagy-related proteins (Beclin-1, LC3- II, and P62) were determined by western blot. Western blot results showed that the H/R group induced higher expression of Beclin-1 and LC 3-II than the control group. Beclin-1 and LC3-II expression levels were significantly lower than those in the H/R group after the use of autophagy inhibitor 3-methyladenine (3-MA) (Figures 2(a)–2(d)). To separately evaluate the effect of H/R and propofol treatment on autophagosomes and autolysosomes, we transfected HUVECs with Ad-mCherry-GFP-LC3B and analyzed the cells via fluorescence microscopy [42, 43]. In the acidic environment of lysosomes, GFP loses its fluorescence, whereas monomeric red fluorescent protein (mCherry) retains its fluorescence. Thus, green LC3 puncta mainly indicate autophagosomes, whereas red LC3 puncta indicate both autophagosomes and autolysosomes in individual images. Red puncta were overlaid with green puncta and appeared as yellow in the merged images, indicating autophagosomes, with free red puncta in the merged images indicating autolysosomes [44]. As shown in Figure 2(e), when HUVECs was treated by H/R, red and yellow puncta markedly accumulated in cells. When 3-MA was used to inhibit autophagy, numerous yellow spots appeared in HUVECs cells. When we added propofol (100 *μ*mol/L) to H/R-treated cells, we could see a small number of yellow spots in the cells. These results demonstrate that H/R treatment activated autophagic flux in HUVECs. However, propofol inhibits autophagic flow formation in vitro H/R model.

We also found that propofol downregulated the level of Beclin-1 and LC3-II induced by H/R in a concentration-depending manner when the concentration of propofol was between 25 and 100 *μ*mol/L but tended to further enhance H/R-induced increases of Beclin-1 and LC3-II at the concentration of 150 *μ*M. Using a western blotting analysis, we showed that excessive propofol has side effects (Figures 2(f)–2(h)). Of note, Beclin-1 and LC3-II levels in the 100 *μ*mol/L propofol group are remarkably lower than in the untreated H/R group and other propofol groups, suggesting that 100 *μ*mol/L propofol may effectively repress hypoxia reoxygenation-induced autophagy (Figures 2(f)–2(h)).

We then investigated the expression of Beclin-1 in human heart samples from the propofol group and the control group. As shown in Figures 2(i) and 2(j), Beclin-1 level in T_3_ of the propofol group was significantly lower than that in T_3_ of the control group (*p* < 0.05, T_1_: 15 min before cardiopulmonary bypass, T_2_: 15 min after cardiac arrest, and T_3_: 10 minutes after heart restated beating). Moreover, Beclin-1 in the propofol group dramatically decreased at T_3_ than T_2_, while the expression of Beclin-1 did not significantly change in the control group (*p* > 0.05). These results demonstrate that propofol inhibits autophagy in H/R (I/R) models in vitro or in vivo.

### 3.2. Propofol Induces miR-30b Expression in H/R (I/R) Models In Vitro or In Vivo

In the previous studies, in order to study the potential miRNAs that may play a protective function in the protective effect on H/R injury in HUVECs, we determined the miRNA expression profile in HUVECs through miRNA microarray analysis. The study found that the expressions of hsa-miR-30b, hsa-miR-20b, hsa-miR-196a, and hsa-miR-374b were upregulated in propofol group after H/R treatment, while hsa-let-7e and hsa-miR-15b showed an opposite expression pattern, which was in agreement with the result of microarray hybridization [7].

**Figure 3:**
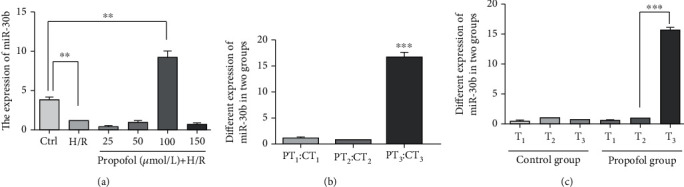
Propofol induces miR-30b expression in H/R (I/R) models in vitro or in vivo. (a) The expression of miR-30b in each group by real-time PCR. ^∗∗^*p* < 0.01, vs. the control group. (b) The different expression of miR-30b between the control group and the propofol group by real-time PCR. ^∗∗∗^*p* < 0.001, T_3_ in the propofol group vs. T_3_ in the control group. (c) The different expression of miR-30b in each group by real-time-PCR. ^∗∗∗^*p* < 0.001, T_3_ in the propofol group vs. T_2_ in the propofol group.

To investigate the molecular mechanism by which propofol exerts its functions in protecting cells, we examined the expressions of microRNA-30b *in vitro*. HUVECs were treated with different concentrations of propofol (0–150 *μ*mol/L) in reoxygenation episode for 4 hours after 12 hours hypoxia. Real-time PCR was used to examine the expression of miR-30b. The endogenous miR-30b in 100 *μ*mol/L propofol group (9.13 ± 1.38%, *p* < 0.01 vs. the control group) increased remarkably than in H/R group (*p* < 0.01 vs. the control group) and other propofol groups (0.3 ± 0.24%, 0.84 ± 0.50%, and 0.62 ± 0.38%, respectively) (Figure 3(a)).

We then investigated the expression of miR-30b in human heart samples from the propofol group and the control group. The expression of miR-30b between the two groups was significantly different at T_3_ (*p* < 0.001) (Figure 3(b)). By intragroup comparison, miR-30b increases dramatically at T_3_ compared with T_2_ within the propofol group, revealing that the expression of miR-30b may be effectively induced by propofol only at T_3_ (Figure 3(c)).

Together, these data indicate that propofol inhibited autophagy and induced miR-30b expression in both in vitro and in vivo H/R (I/R) models.

### 3.3. Beclin-1 Is a Direct Target of miR-30b

**Figure 4:**
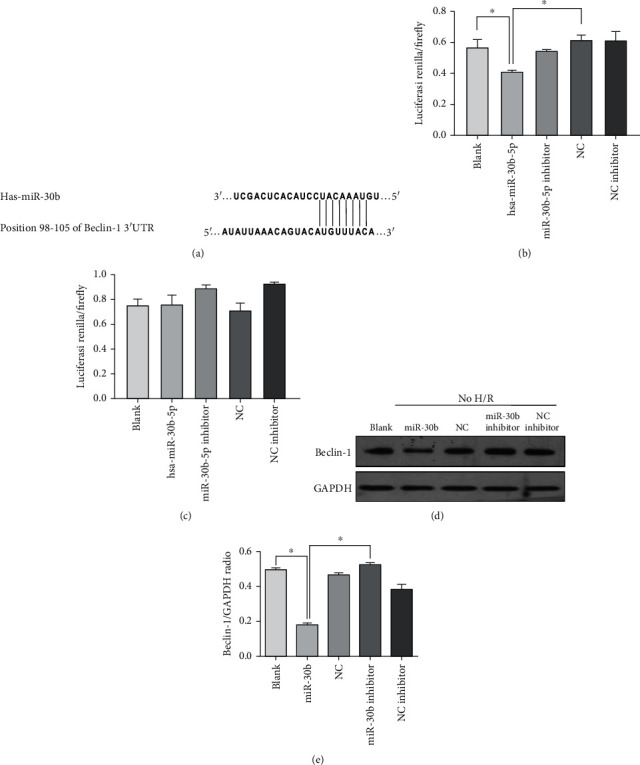
Beclin-1 is a direct target of miR-30b. (a) Diagram of the miR-30b pairing sequence in 3′UTR of Beclin-1. The matched base pairs are connected by a vertical line. Beclin-1 3′UTR relevant fragments were inserted downstream of the firefly luciferase gene of the pmirGLO vector. (b) The different expression of relative fluorescence intensity in each group by dual-luciferase reporter gene assay. The 293T cells were cotransfected with 3′UTR Beclin-1 plasmid and each different miRNA, and the relative fluorescence intensity of the samples was calculated. ^∗^*p* < 0.05, vs. the hsa-miR-30b-5p group. *p* > 0.05, the NC group vs. the hsa-miR-30b-5p inhibitor group. *p* > 0.05, the NC inhibitor group vs. the hsa-miR-30b-5p inhibitor group. (c) The different expression of relative fluorescence intensity in each group by dual-luciferase reporter gene assay. The 293T cells were cotransfected with mut3′UTR Beclin-1 plasmid and each different miRNA, and the relative fluorescence intensity of the samples was calculated. *p* > 0.05, the NC group vs. the hsa-miR-30b-5p group. *p* > 0.05, the blank group vs. the hsa-miR-30b-5p group. (d–e) The expression of autophagy-related protein Beclin-1 in blank and transfecting cells with miR-30b, miR-30b control, miR-30b inhibitor, and miR-30b inhibitor control groups without H/R treatment. ^∗^*p* < 0.05, vs. the hsa-miR-30b-5p group. Mean ± SD of 3 independent trials. Abbreviations: miR-30b: hsa-miR-30b-5p; NC: the negative control.

To uncover the molecular mechanism of autophagy inhibition mediated by miR-30b, we searched for all autophagy genes containing potential miR-30b recognition sites in their 3′UTRs using multiple bioinformatics tools (Luciferase assay, Computational algorithms Miranda, and TargetScan). Interestingly, the core autophagy gene *beclin-1* was identified as a potential miR-30b target (Figure 4(a)). The predicted binding sites between miR-30b and the *beclin-1* 3′UTR is shown in Figure 4(a). Beclin-1 3′UTR contains a single 8-mer seed match to miR-30b at nt. 98-105.

To confirm the in silico-based predictions, we next examined the ability of miR-30b to regulate *beclin-1*. We cloned 724 base-pair 3′UTR fragments from *beclin-1* to a dual-luciferase reporter system and tested the ability of miR-30b to regulate the reporters. The 293T cells were cotransfected with 3′UTR Beclin-1 plasmid and each different miRNA, and the relative fluorescence intensity of the samples was calculated. As shown in Figures 4(b) and 4(c), hsa-miR-30b-5p can affect the fluorescence activity of Beclin-1 gene by binding to its 3′UTR (*p* < 0.05 vs. the blank group), and hsa-miR-30b-5p could not affect the fluorescence activity of Beclin-1 gene by binding to its mut3′UTR (*p* > 0.05 vs. the blank group). The 3′UTR of the beclin-1 gene responded markedly to miR-30b overexpression relative to the blank group.

Then, we performed western blotting analysis of extracts from miRNA-transfected cells or blank cells using anti-Beclin-1-specific antibodies. Overexpression of miR-30b resulted in a potent downregulation of Beclin-1 protein level in HUVECs (*p* < 0.05 vs. the blank group). Conversely, the introduction of the miR-30b inhibitor group resulted in an increase in Beclin-1 protein level compared to the blank group and the miR-30b group (Figures 4(d) and 4(e)).

### 3.4. miR-30b Suppresses H/R-Induced Autophagy Activation

**Figure 5:**
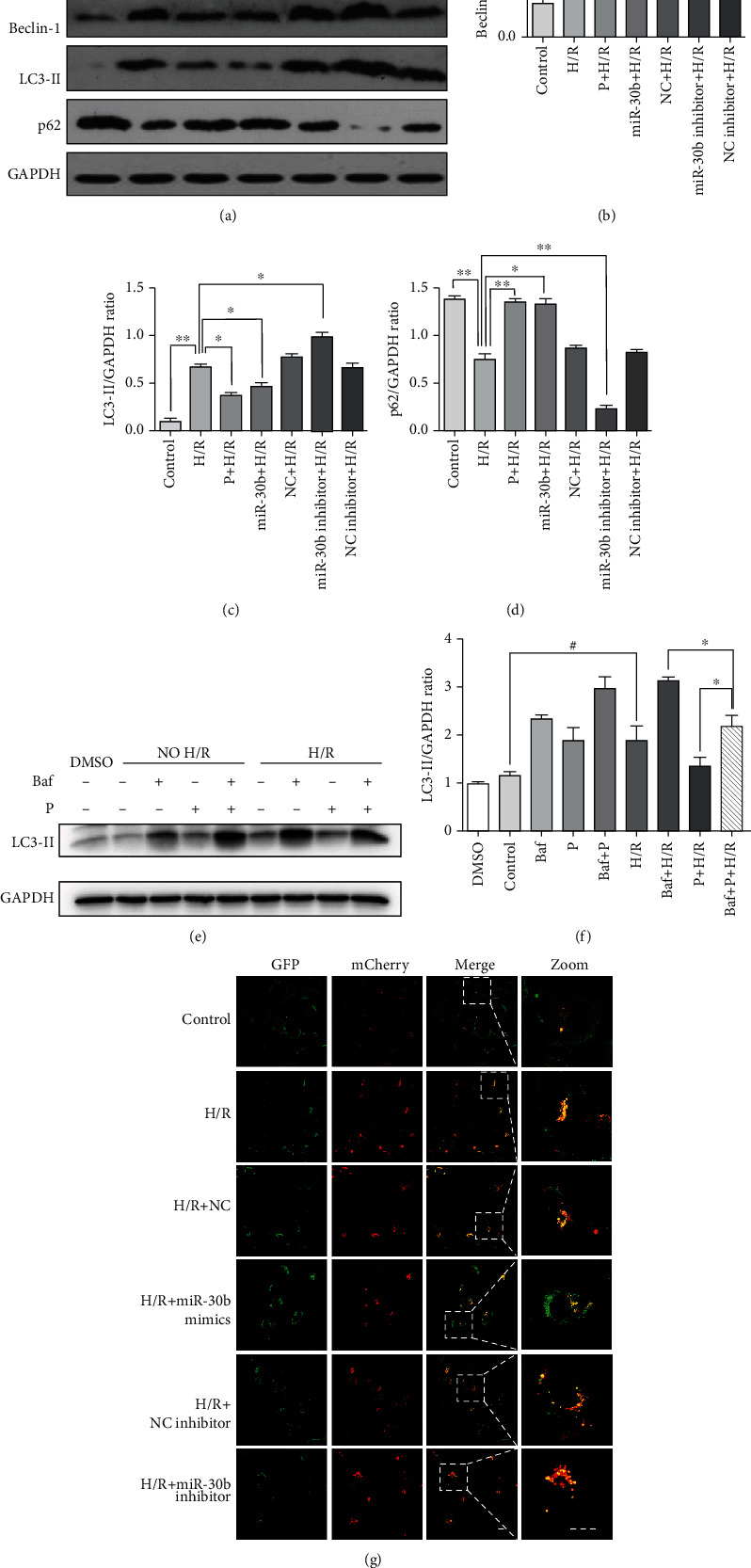
miR-30b suppresses H/R-induced autophagy activation. (a–d) The HUVECs were transfected with each different miRNA. The different expressions of Beclin-1, LC3-II, and p62 in each group. (e–f) The expressions of GAPDH and LC3-II in the normal group, the H/R group, and 100 *μ*mol/L propofol or 10 *μ*M bafilomycin A1 H/R treatment group. ^∗^*p* < 0.05, vs. the Baf+p+H/R group. ^#^*p* < 0.05, the control group vs. the Baf+p+H/R group. (g) The HUVECs were transfected with Ad-mCherry-GFP-LC3B and observed under a fluorescence microscope. Yellow puncta: red puncta overlaid with green puncta and red puncta: autolysosomes. Scale bar = 20 *μ*m. Mean ± SD of 3 independent trials. Abbreviations: 3-MA: 3-methyladenine; HUVECs: human umbilical vein endothelial cell; P: propofol; H/R: hypoxia/reoxygenation; miR-30b: hsa-miR-30b-5p; NC: the negative control; Baf: bafilomycin A1.

Next, we investigated the role of miR-30b in H/R-induced autophagy. Compared with other groups, Beclin-1 and LC3-II protein expressions were significantly decreased in the 100 *μ*mol/L propofol-treated group or miR-30b-transfected group under hypoxia and reoxygenation, while p62 expression was increased under such conditions (Figures 5(a)–5(d)). To test the effects of endogenous miR-30b inhibition on autophagy, we used miR-30b inhibitor to analyze autophagy under H/R conditions. Beclin-1 and LC3-II increased significantly through the inhibition of endogenous miR-30b by inhibitor compared with the H/R group (Figures 5(a)–5(d)).

To clarify the specific role of propofol in the inhibition of autophagy and distinguish the induction of autophagy from the damage of autophagy flux, the medium was added with bafilomycin A1 and/or propofol after 12 hours of hypoxia. The LC3-II level in the group treated with both propofol and bafilomycin after hypoxia was significantly decreased compared with that in the group treated with bafilomycin A1 alone after hypoxia, indicating that propofol inhibited autophagy (Figures 5(e) and 5(f)). The Ad-mCherry-GFP-LC3B is used to observe alterations in autophagic flux, as green fluorescence protein (GFP) but not RFP is quenched upon autophagic delivery of the fusion protein to the acidic lysosome; nonacidic autophagic vacuoles appear as yellow puncta. As shown in Figure 5(g), when HUVECs was treated by H/R, red and yellow puncta markedly accumulated in cells. In cells treated with H/R superimposed with has-miR-30b inhibitor, macula increased. Compared to the H/R group, miR-30b overexpression significantly decreased both red and yellow puncta (Figure 5(g)), suggesting that miR-30b inhibits autophagic flux. These findings confirm that miR-30b inhibits H/R-induced autophagy activation.

### 3.5. MirRNA-30b Represses H/R-Induced Autophagy and Promotes Cell Survival

**Figure 6:**
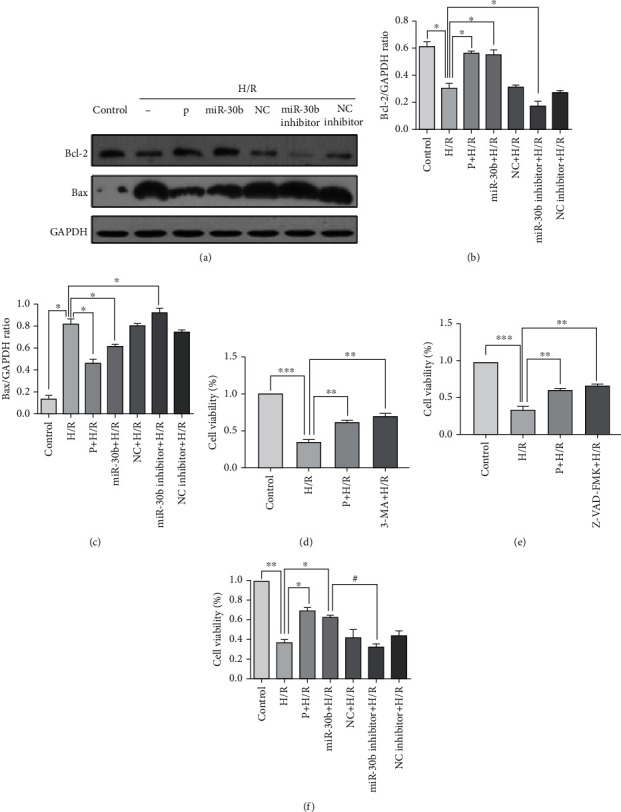
MicroRNA-30b represses H/R induced autophagy and promotes cell survival. The cells were transfected with miR-30b, NC, miR-30b inhibitor, and NC inhibitor and treated with 12 h of hypoxia and 4 h of reoxygenation. (a–c) The expression of Bcl-2, Bax in control, hypoxia reoxygenation injury group (H/R), 100 *μ*mol/L propofol posthypoxia treatment group (P), and transfecting microRNAs groups were examined. ^∗^*p* < 0.05, vs. the H/R group. (d–f) Cell viability was determined by CCK-8 assay, as previously described. Values are presented as the percentage of viable cells. ^∗^*p* < 0.05, compared with the H/R group. ^∗∗^*p* < 0.01, compared with the H/R group. ^∗∗∗^*p* < 0.001, compared with the H/R group.

Our above study showed that miR-30b suppresses H/R-induced autophagy activation. Ultrastructure of cardiomyocytes treated with H/R was observed by TEM. Cell membrane of the control group was intact, and the morphology of apoptosis and autophagy was less. In H/R group, nuclear membrane was ruptured, chromatin was concentrated, autophagy vacuoles were increased, and mitochondria were swollen [45]. To further examine the other specific roles of miR-30b in the process of H/R induced injury of HUVECs, we detected the level of apoptosis relevant proteins. As shown in Figures 6(a)–6(c), apoptotic related protein Bax was upregulated, while antiapoptotic protein Bcl-2 was downregulated in the H/R group. The effect was exacerbated in miR-30b inhibitor group when the function of endogenous miR-30b had been blocked by miR-30b inhibitor. However, propofol (100 *μ*mol/L) and miR-30b reversed the trend, by downregulating Bax and upregulating Bcl-2 (Figures 6(a)–6(c)), suggesting that miR-30b may also play a role in inhibiting apoptosis.

To further examine the specific role of autophagy and apoptosis in the process of H/R-induced injury of HUVECs, 3-MA and Z-VAD-FMK were added to the cells. The CCK-8 assay demonstrated that treatment with 3-MA at a concentration of 10 mmol/L significantly alleviated the decrease in cell viability induced by H/R (*p* < 0.01 vs. the H/R group) (Figure 6(d)). The Z-VAD-FMK group had similar results (Figure 6(e)), suggesting a lot of autophagy and apoptosis were induced by H/R. The protective effect of miR-30b on cell viability was also further confirmed by the cell viability assay. After H/R treatment, the cell viability of the H/R group decreased dramatically (36.4 ± 3.3%, *p* < 0.01 vs. the control group), but it was partially restored by the 100 *μ*mol/L propofol group (68.7 ± 3.6%, *p* < 0.05 vs. the H/R group) as well as the has-miR-30b group (62.1 ± 1.2%, *p* < 0.05 vs. the H/R group), while miR-30b inhibitor (31.9 ± 3.0%) aggravated the damage (*p* < 0.05 vs. the H/R+miR-30b group) (Figure 6(f)). These results showed that miR-30b inhibited H/R-induced autophagy and promoted cell survival. And, miR-30b may also play a role in inhibiting apoptosis. More studies are needed to prove the molecular mechanism of miR-30b inhibiting apoptosis.

## 4. Discussion

Ischemia reperfusion is a common situation in clinical settings including electric liver resection, trauma, and transplantation [46]. Autophagy is also widely implicated in a number of heart diseases [47]. The degree of autophagy is mediated by ischemia and is increased during myocardial I/R [48]. Under the condition of cardiac I/R injury, the process of autophagy is activated in response to energy crisis and oxidative stress [49]. However, it has been accepted that autophagy can be a two-edged sword in I/R [49]. A large body of evidence indicates that an unbalanced autophagic response directly results in cell damage and cell death, but its mechanism remains elusive [50–53]. In our study, we found that hypoxia reoxygenation injury may lead to overexpression of Beclin-1 resulting in overactivation of autophagy and decrease cell viability and promotion of cell death.

A large number of studies have shown that sevoflurane treatment has a protective effect on cardiac ischemic tissues [8, 9, 54–56]. Sevoflurane has previously been found to protect endodermic glycocalyx from ischemia-reperfusion-induced degradation [56]. Another research found that sevoflurane posttreatment improved cardiac function after ischemia-reperfusion in rats, which may be related to the improvement of mitochondrial respiratory function after upregulation of HIF-1*α* expression. Sevoflurane can upregulate the expression of HIF-1*α* via the PI3K-Akt-mTOR pathway [54]. Recent results suggest that sevoflurane-pretreated mesenchymal stem cells can promote H/R injury angiogenesis and reduce myocardial I/R injury in HUVECs [55].

Propofol is a protective drug against ischemia-reperfusion injury both in vitro and in vivo. Li et al. indicated that propofol exerts cardioprotection when administered at the early phase of reperfusion. The effect is mediated through decrease in cardiomyocyte apoptosis and NF-*Κ*B nucleus translocation potentially via ERK signaling pathways [57]. Wang et al. found that propofol postconditioning induced long-term neuroprotection and reduced internalization of AMPAR GluR2 subunit in a rat model of focal cerebral ischemia/reperfusion [58]. Propofol inhibits peroxidation scavenging free radicals for antioxidant activity [59–61] and attenuates reperfusion injury by suppressing autophagic cell death, as it may directly suppress autophagy or indirectly modulate the production of other cytotoxic mediators [23]. These cytotoxic mediators include free radicals, glutamate, or calcium, which can alter mitochondrial integrity or trigger autophagy activation [16]. Xia et al. observed that sevoflurane or desflurane anesthesia plus postoperative propofol sedation attenuates myocardial injury after coronary surgery in elderly high-risk patients [62]. Some studies also suggested that synergy of isoflurane preconditioning plus propofol postconditioning may confer superior protection against myocardial I/R in patients compared with an isoflurane or propofol anesthesia regimen alone and that the mechanism of the synergy is related to superoxide anion, NO, and ONOO^−^ [63, 64]. ONOO^−^ at low levels is cardioprotective and can serve as a trigger of ischemia preconditioning [65, 66]. However, ONOO^−^ is detrimental to the heart or cardiomyocytes when its production is increased during reperfusion [67, 68]. Therefore, the protection against myocardial I/R conferred by propofol postconditioning might be attributable to propofol scavenge ONOO^−^ property. Previously, our study also demonstrated that propofol can inhibit excessive autophagy by hypoxia reoxygenation, but the mechanisms need to be further explored [7]. In summary, the purpose of this study was to compare whether posttreatment anesthesia with propofol after ischemia (the propofol group) and posttreatment anesthesia with midazolam after ischemia (the control group) had different effects on the heart during mitral valve surgery on the basis of sevoflurane anesthesia.

In general, propofol was delivered at 25 mL/h in a 70 kg human, which is often sustainable and will not result in significant systemic hypotension. In our *in vitro* cell culture study, propofol was delivered directly at 25, 50, 100, and 150 *μ*M concentrations. The clinically relevant concentration of propofol was 2-11 *μ*g/mL (approximately 10-62 *μ*M), so the concentration of 25, 50, 100, and 150 *μ*M was clinically relevant [69–72]. We select 150 *μ*M to compare the effects in this extreme concentration with clinic relevant one. Moreover, using the extreme value of drug concentration, we can better reveal the mechanism of drug action in the in vitro model. Similar concentrations had been reported having protective effect on hypoxia-induced apoptosis in alveolar epithelial type II cells. We also found in our previous paper that 100 *μ*M concentration is very effective to protect cells in response to hypoxia/reoxygenation [7].

Herein, we established a molecular link between propofol postconditioning and its anti-I/R-induced cell injury. Overactivation of autophagy by I/R can be effectively suppressed by administration of an appropriate dose of propofol in vitro and in human patients, which is at least in part due to the enhanced expression of miR-30b and downregulating of Beclin-1. Interestingly, the core autophagy gene is a direct target of miR-30b. Of note, miR-30b is dramatically upregulated at the onset of reperfusion in the presence of propofol (T_3_ with propofol) in patients undergoing open heart surgery.

Our study established a novel relationship between the propofol-regulated microRNA and autophagy. We show that propofol increases the expression of miR-30b to inhibit H/R- or I/R-induced overexpression of Beclin-1, thereby inhibiting excessive autophagy. Interestingly, mir-30b may play a protective role in propofol inhibition of both excessive autophagy and apoptosis. Therefore, miR-30b may be potentially regarded as a biomarker is assessing or estimating the effectiveness of propofol in preventing H/R- or I/R-induced injury.

## 5. Conclusions

In conclusion, this study experimentally demonstrated the cardioprotective role of propofol during anesthesia. The cardioprotective effects of propofol may be mediated by the upregulates microRNA-30b to repress excessive autophagy via targeting Beclin-1 under H/R condition.

## Figures and Tables

**Table 1:** Patients characteristics.

	Control group	Propofol group	*p* value
Age (years)	50 ± 11.93	45 ± 8.65	0.15
Sex			
Male number	6	5	0.72
Female number	14	15	
Height (cm)	158 ± 8.62	159 ± 7.85	0.83
Weight (kg)	54 ± 8.24	54 ± 7.15	0.91
Body surface area (m^2^)	1.58 ± 0.2	1.53 ± 0.12	0.66
Fasting blood glucose (mmol/L)	5.47 ± 1.47	6.47 ± 2.26	0.19
ALT (U/L)	20.41 ± 10.70	20.43 ± 12.90	0.96
AST (U/L)	20.28 ± 4.44	20.70 ± 6.04	0.79
Urea (mmol/L)	5.98 ± 2.09	5.09 ± 1.32	0.14
Scr (*μ*mol/L)	81.20 ± 19.70	82.71 ± 22.35	0.86
SUA (*μ*mol/L)	364.47 ± 59.58	363.23 ± 98.94	0.96
eGFR (ml/min)	75.69 ± 13.70	79.94 ± 18.75	0.49
CHOL (mmol/L)	4.95 ± 0.87	4.22 ± 1.15	0.06
TG (mmol/L)	1.62 ± 0.78	1.52 ± 0.91	0.70
HDL-CH (mmol/L)	1.14 ± 0.31	1.04 ± 0.23	0.34
LDL-CH (mmol/L)	3.07 ± 0.84	2.60 ± 1.07	0.20

Abbreviations: SD: standard deviation; ALT: alanine aminotransferase; AST: glutamate aminotransferase; Scr: serum creatinine; SUA: serum uric acid; eGFR: estimated glomerular filtration rate; CHOL: cholesterol; TG: triglyceride; HDL-CH: high density lipoprotein cholesterol; LDL-CH: low density lipoprotein cholesterol. Data are mean ± SD except for gender.

**Table 2:** Perioperative adverse reactions.

	Control group (*n* = 20)	Propofol (*n* = 20)	*p* value
Hypotension	2	1	0.55
Bradycardia	3	1	0.29
Respiratory	0	0	
Nausea and vomiting	0	0	

**Table 3:** Perioperative data.

		Control group	Propofol group	*p* value
CPB time (min)		90.19 ± 15.26	87.65 ± 20.93	0.48
Aortic cross-clamp time (min)		60.41 ± 9.54	58.56 ± 6.84	0.11
Postoperative mechanical ventilation (hours)		19.13 ± 4.4	17.71 ± 3.53	0.31
Operative time (min)		174.5 ± 22.2	175.29 ± 26.9	0.93
ICU stay (days)		1.79 ± 0.57	1.57 ± 0.39	0.21
Hospitalization (days)		12.63 ± 2.09	11.53 ± 1.66	0.11
Automatic heart resuscitating number		12/20	15/20	0.31
Heart rate (beat/min)	T_0_	81.88 ± 14.71	83.53 ± 11.98	0.72
	T_1_	72.44 ± 14.63	77.82 ± 12.49	0.26
	T_3_	87.63 ± 9.22	79.18 ± 11.24	0.03
	T_4_	90.38 ± 10.57	80.24 ± 13.64	0.02
	T_5_	94.88 ± 10.4	88.59 ± 11.4	0.11
MAP (mmHg)	T_0_	80.15 ± 9.83	80.4 ± 7.09	0.93
	T_1_	72.19 ± 7.41	73 ± 9.62	0.79
	T_2_	48.25 ± 7.53	50.88 ± 6.78	0.30
	T_3_	69.56 ± 7.24	63.76 ± 5.74	0.02
	T_4_	81.13 ± 6.45	75.12 ± 4.73	≤0.001
	T_5_	80.33 ± 7.82	76.39 ± 6.44	0.12

Abbreviations: SD: standard deviation; CPB: cardiopulmonary bypass; ICU: intensive care unit; MAP: mean arterial pressure; T_0_: before anesthesia induction; T_1_: 15 min before cardiopulmonary bypass; T_2_: 15 min after cardiac arrest; T_3_: 10 minutes after heart restated beating; T_4_: 1 h after heart restated beating; T_5_: 24 h after operation. Data are mean ± SD except for automatic heart resuscitating number.

## Data Availability

The data used to support the findings of this study are included within the article.

## Abbreviations

H/R: Hypoxia/reoxygenation
P-PostH: Propofol posthypoxia treatment
HUVECs: Human umbilical vein endothelial cell
miR-30b: MicroRNA-30b
P: The propofol group
Ctrl or C: The control group
I/R: Ischemia-reperfusion
ROS: Reactive oxygen species
miRNAs: MicroRNAs
DMEM: Dulbecco's modified Eagle's medium
SD: Standard deviation
ALT: Alanine aminotransferase
AST: Glutamate aminotransferase
Scr: Serum creatinine
SUA: Serum uric acid
eGFR: Estimated glomerular filtration rate
CHOL: Cholesterol
TG: Triglyceride
HDL-CH: High density lipoprotein cholesterol
LDL-CH: Low density lipoprotein cholesterol
CVP: Central venous pressure
BIS: Bispectral index
HR: Heart rate
CPB: Cardiopulmonary bypass
ICU: Intensive care unit
MAP: Mean arterial pressure
T_0_: Before anesthesia induction
T_1_: 15 min before cardiopulmonary bypass
T_2_: 15 min after cardiac arrest
T_3_: 10 minutes after heart restated beating
T_4_: 1 h after heart restated beating
T_5_: 24 h after operation
TNT-HS: Sensitive troponin T
CK-MB: Creatine kinase isoenzyme muscle/brain
miR-30b: hsa-miR-30b-5p
NC: The negative control
3-MA: 3-Methyladenine
Baf: Bafilomycin A1.

## Supplementary Materials

Supplementary Materials Screenshot of the web version of the detailed clinical study plan.
