# Supplementary figures and images for: Propofol Upregulates MicroRNA-30b to Inhibit Excessive Autophagy and Apoptosis and Attenuates Ischemia/Reperfusion Injury In Vitro and in Patients
Source: Oxid Med Cell Longev. 2022 Mar 30;2022:2109891. doi: 10.1155/2022/2109891 (PMC8986434; doi:10.1155/2022/2109891)

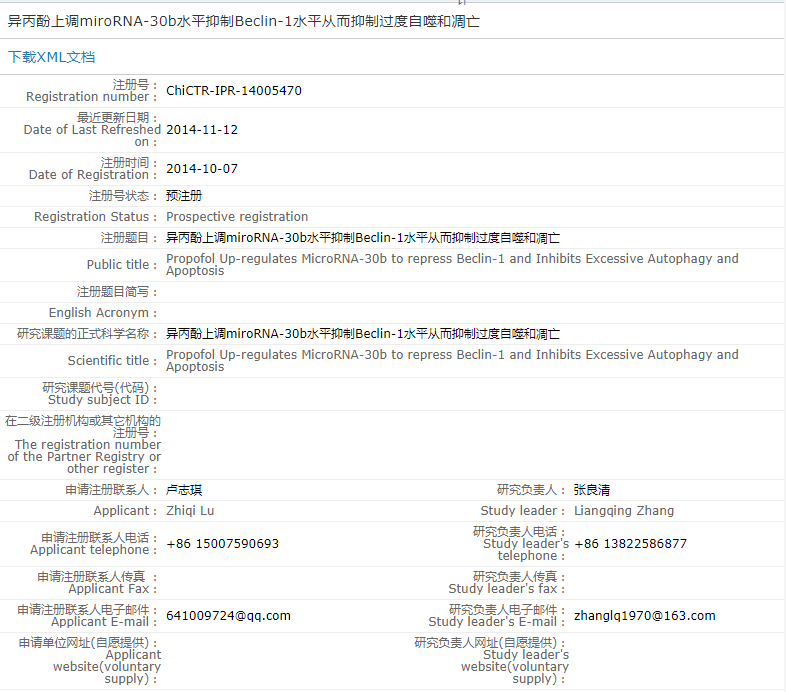

Supplement: Supplementary Materials — Screenshot of the web version of the detailed clinical study plan. [file 2109891.f1.zip › Protocols in the Clinical Sciences-1 (1).png]

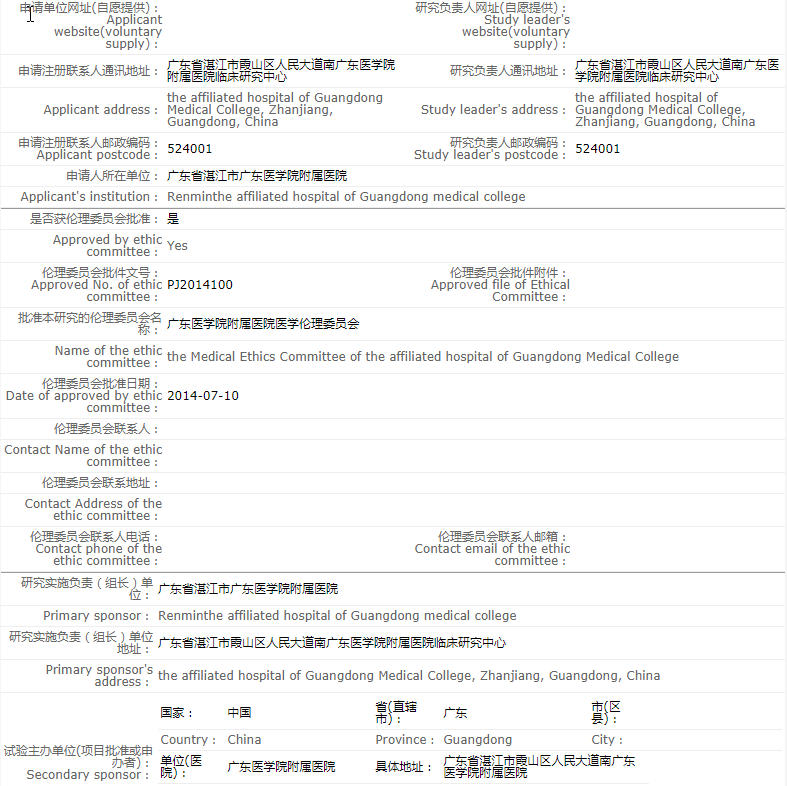

Supplement: Supplementary Materials — Screenshot of the web version of the detailed clinical study plan. [file 2109891.f1.zip › Protocols in the Clinical Sciences-2 (1).png]

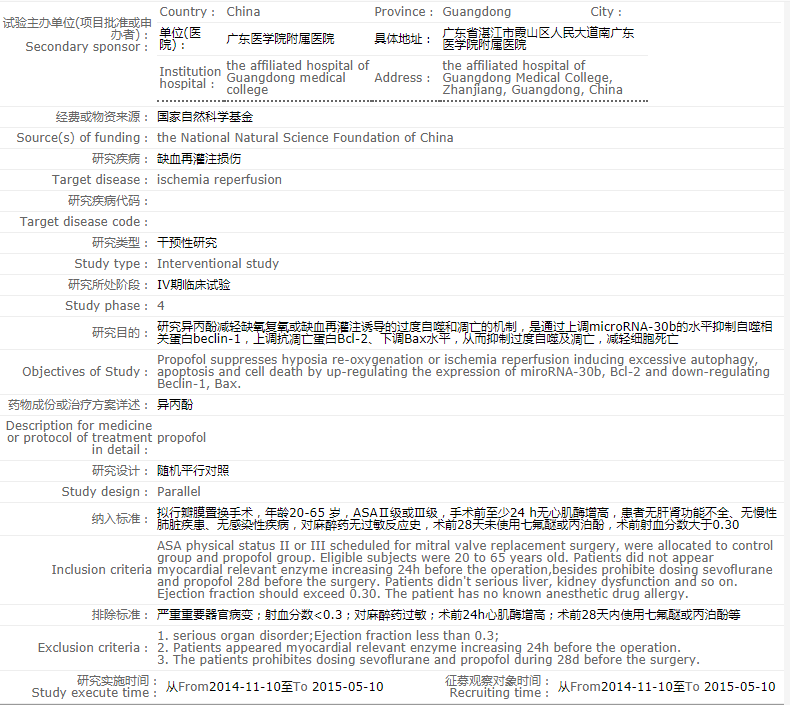

Supplement: Supplementary Materials — Screenshot of the web version of the detailed clinical study plan. [file 2109891.f1.zip › Protocols in the Clinical Sciences-3 (1).png]

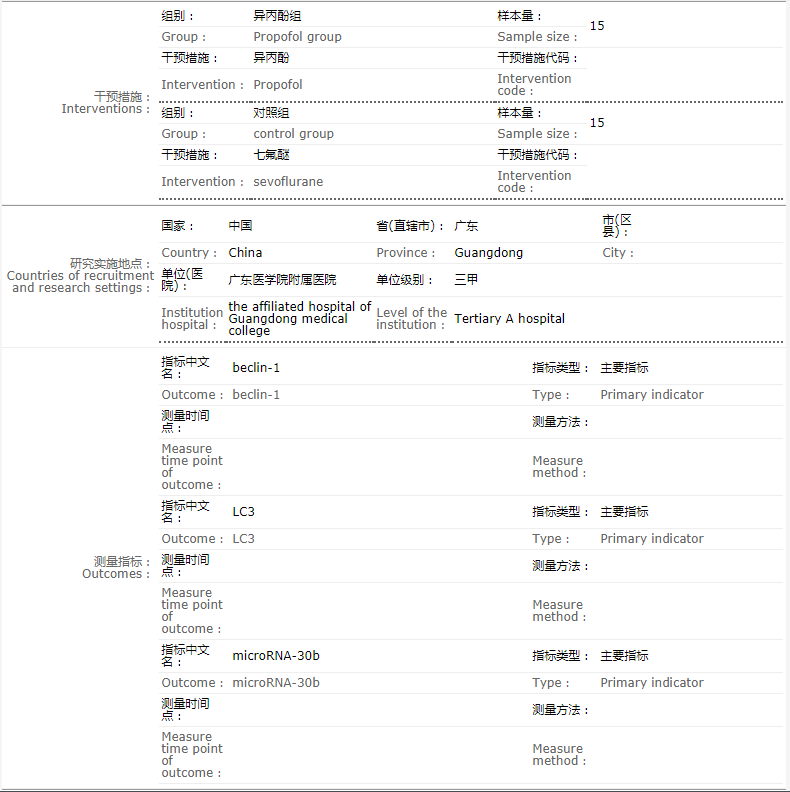

Supplement: Supplementary Materials — Screenshot of the web version of the detailed clinical study plan. [file 2109891.f1.zip › Protocols in the Clinical Sciences-4 (1).png]

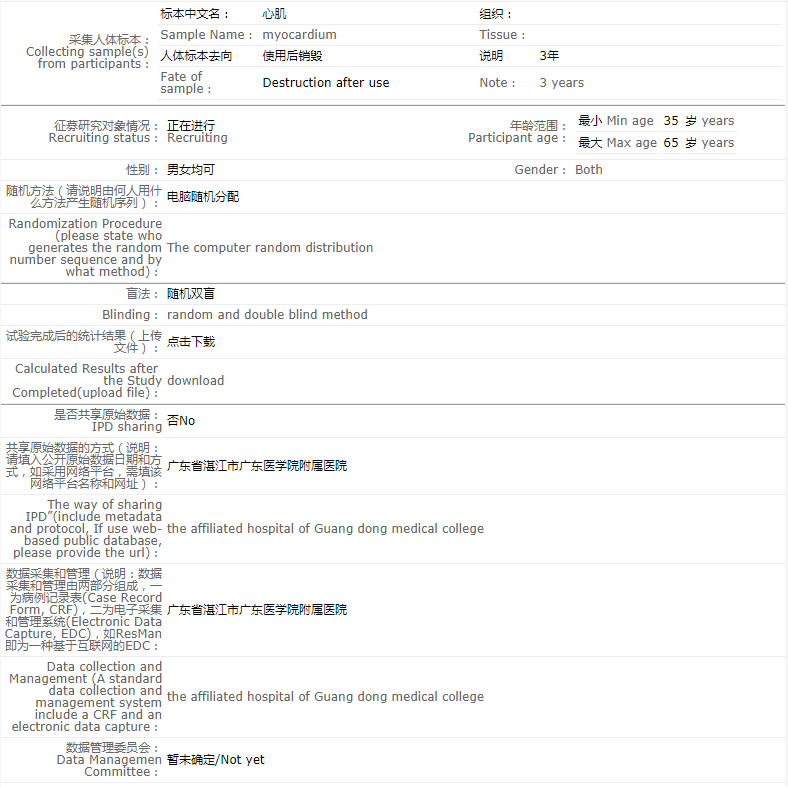

Supplement: Supplementary Materials — Screenshot of the web version of the detailed clinical study plan. [file 2109891.f1.zip › Protocols in the Clinical Sciences-5 (1).png]
